# Tertiary Lymphoid Structures in Tuberculosis: Persistence, Protection, and Pathology

**DOI:** 10.1111/imr.70055

**Published:** 2025-08-15

**Authors:** Tiphaine M. N. Camarasa, Lorenzo Iseppi, David Schreiner, Carolyn G. King

**Affiliations:** ^1^ Infection Immunology Laboratory, Department of Biomedicine University of Basel Basel Switzerland

**Keywords:** B cells, iBALT, respiratory diseases, T cells, TLS, tuberculosis

## Abstract

Tuberculosis (TB), caused by *Mycobacterium tuberculosis* (Mtb), is a major public health burden responsible for over a million deaths each year. A deeper understanding of the mechanisms that balance protective immunity and immunopathology is essential for developing more effective therapeutics. This review focuses on the dynamic interplay between CD4^+^ T cells and B cells in the lung, with an emphasis on their interactions in tertiary lymphoid structures (TLS). TLS are immune cell aggregates that arise in inflamed, nonlymphoid tissues, range from loosely to highly organized clusters, and serve as localized hubs for immune cell interaction, activation, and diversification. Drawing on insights from other disease contexts, including infections, cancer, and chronic inflammatory conditions, we examine the molecular signals and cellular interactions involved in TLS formation, maintenance, and function during Mtb infection. Additionally, we explore the anatomical and functional integration of TLS with the lymphatic and vascular systems, and how this spatial organization may influence bacterial persistence and dissemination. Clarifying the functional role of TLS in TB—whether they support protective immunity, contribute to lung pathology, or both—could inform novel approaches to modulate local immune responses and improve TB disease outcomes.

## 1 Introduction

Tuberculosis (TB) is caused by *Mycobacterium tuberculosis* (Mtb), a bacterium transmitted through the air when an infected person coughs or sneezes [1]. Although antibiotic therapy has been life‐saving, TB remains the leading cause of death from a single infectious agent, affecting approximately a quarter of the world's population and killing over a million people every year [2]. TB currently has a disproportionate impact on the world's most vulnerable communities, including those living in poverty, in overcrowded conditions, and with limited access to health care [3, 4]. Global efforts to combat TB are hampered by the emergence of multidrug‐resistant strains and stagnation in the TB drug pipeline, as well as the limited efficacy of the only licensed vaccine, Bacille Calmette‐Guérin (BCG), in preventing disease in adults living in TB‐endemic areas. Moreover, host‐directed therapies and immunomodulatory approaches are rarely applied due to our incomplete understanding of TB immunopathogenesis. Approximately 5% of infected individuals go on to develop active disease within the first 2 years, while an additional 5% may do so later in life, even after years of latent infection [5]. Importantly, TB is now increasingly recognized as a spectrum of disease, ranging from latent infection to subclinical and active disease, with individuals potentially progressing or regressing along this continuum, depending on host immunity and comorbidities (e.g., HIV, diabetes) [6, 7, 8].

Following inhalation, Mtb initially infects alveolar macrophages, which are resident in the airways and provide a permissive intracellular niche for bacterial survival [9, 10]. The bacilli then disseminate into the lung interstitium, leading to the recruitment of myeloid cells and the formation of a multicellular complex called a granuloma at the site of infection [11]. In the early stages after infection, Mtb is contained within granulomas, which act as physical and immunological barriers to limit bacterial spread. However, this containment also delays Mtb transport to the draining lymph nodes, impeding engagement of the adaptive immune response [10, 12]. T‐cell priming in the lymph node depends on the transport of bacteria by migratory dendritic cells and inflammatory monocytes—a process that is considerably delayed, leading to antigen‐specific T‐cell responses that peak several weeks later [13]. As the infection progresses, recruitment of lymphocytes to the lung promotes further granuloma maturation. Granulomas are spherical structures consisting of a central core of infected macrophages and necrotic debris surrounded by a rim of lymphocytes, including CD4 and CD8 T cells and B cells [14]. Additional immune and stromal cell types, including NK cells, neutrophils, dendritic cells, and fibroblasts, are also present. While the granuloma has the potential to contain Mtb and prevent systemic dissemination, it also offers a protective niche that allows Mtb to survive in a dormant state for years [15]. Importantly, Mtb takes advantage of the host immune response at various stages of its life cycle to establish intracellular niches, control cell death pathways, impair antigen‐presentation, and induce tissue damage for transmission [10]. Although the lung is the primary entry point for Mtb, emerging evidence suggests that the infection may disseminate through hematogenous spread and lymphatic routes, seeding distal sites such as the liver, spleen, and bone marrow, even before the onset of overt pulmonary disease. Therefore, what is traditionally recognized as localized pulmonary TB may in some cases reflect a later‐stage re‐localization or reactivation of a more broadly systemic infection [16, 17].

At later stages of Mtb infection, the adaptive immune response becomes increasingly important to both containment and pathogenesis. Recruited lymphocytes fuel granuloma maturation and contribute to the emergence of a more structurally compartmentalized immune environment, including the formation of tertiary lymphoid structures (TLS), also called inducible bronchus‐associated lymphoid tissues (iBALT) in the lung. Understanding how these microenvironments are organized, how they develop and mature over time, and how they influence immune function is essential for guiding the development of new therapeutic strategies and vaccines. In the following sections, we will examine the individual and cooperative roles of CD4 T cells and B cells in the context of Mtb infection and explore how their interactions within TLS shape trajectories for host protection and disease.

## 2 CD4 T Cells and Tuberculosis

CD4 T cells play an essential role in controlling Mtb infection and preventing disease progression [18, 19, 20]. This is clearly demonstrated in HIV‐infected individuals, where CD4 T cell depletion significantly increases their susceptibility to active TB [21, 22, 23]. Similar findings are observed in experimental models where depletion of CD4 T cells in both mice and nonhuman primates (NHPs) leads to elevated bacterial burden and decreased survival [24, 25, 26, 27, 28, 29]. While CD4 T cells are indispensable for immunity, their delayed recruitment to the lung likely limits their ability to fully sterilize Mtb. Type 1 T helper (Th1) cells are the canonical CD4 T cell subset associated with protective immunity to Mtb. These cells secrete IFNγ and TNFα, which activate macrophages to increase bacterial phagocytosis and killing. Accordingly, individuals with inborn errors in the IL‐12/IFNγ axis are highly susceptible to TB, while mice genetically deficient in T‐bet, TNFα, IFNγ, or IL‐12 fail to control bacterial growth [30, 31, 32, 33, 34, 35, 36, 37, 38, 39]. Nevertheless, IFNγ alone is not a reliable correlate of protection, further illustrated by vaccine trials where targeting the differentiation of a memory Th1 response did not translate to protection [26, 40, 41, 42, 43, 44, 45]. This suggests that additional CD4 T cell subsets are necessary to orchestrate host protection. A recent study using scRNAseq to characterize lung T‐cell heterogeneity and dynamics in Mtb‐infected mice reported the differentiation of Th17 cells, tissue‐resident memory T cells (T_RM_‐like), T follicular helper cells (Tfh‐like), and regulatory T cells (Treg) [46]. In this study, the authors emphasized the limited development of polyfunctional T cells with the capacity to secrete multiple effector cytokines and increased numbers of “dysfunctional” CD4 T cells with an exhausted transcriptional signature at later time points after infection.

### 2.1 Th17 Cells

Th17 cells and their hallmark cytokine IL‐17 have recently emerged as important contributors to TB immunity, particularly in the context of mucosal vaccination [47]. Both BCG vaccination and Mtb infection elicit lung‐localized Th17 cells [48, 49, 50, 51, 52]. IL‐17 promotes the production of chemokines (e.g., CXCL1, CXCL5, CCL20) by lung epithelial cells and fibroblasts, resulting in the recruitment of neutrophils, Th1 cells, and the formation of iBALT [53, 54, 55, 56, 57, 58]. Mucosal adjuvants that foster IL‐17 production have also been shown to induce the accumulation of lung Th17 cells, which facilitate the positioning of CXCR5^+^ T cells and B cells within iBALT, thereby fostering early macrophage activation and improved bacterial control following Mtb challenge [59]. The use of liposomal adjuvants to induce combined Th1/Th17 memory responses demonstrated efficacy, with increased vaccine‐specific antibody responses and protection against Mtb aerosol challenge in mice [60]. Overall, a hybrid Th1/Th17 immune profile is increasingly associated with improved outcomes, particularly following mucosal BCG vaccination [52, 61, 62, 63, 64].

### 2.2 T Follicular Helper (Tfh) Cells

The role of Tfh cells in TB has gained significant attention in recent years. Tfh cells support humoral immunity by aiding B‐cell maturation and antibody class switching within germinal centers. Mtb‐specific antibodies can be detected in both Mtb‐infected and ‐exposed humans, with antibody titers generally increasing with the severity of disease [65, 66, 67]. In a Ugandan cohort of TB “resisters” (highly exposed to Mtb but persistently negative for the tuberculin skin test), non‐IFNγ‐producing Mtb‐specific T cells were characterized by elevated expression of CD40L, a hallmark of Tfh function [68]. These resisters also showed increased antibody avidity and class‐switched IgG, suggesting a role for Tfh‐driven humoral responses. In murine models, lung‐resident Tfh‐like cells expressing CXCR5, PD‐1, ICOS, and Bcl6, the canonical Tfh transcription factor, are required for bacterial control and implicated in T‐cell positioning in the lung parenchyma [69, 70, 71].

### 2.3 Tregs

The role of Tregs in TB remains complex and context‐dependent [72]. While Tregs can limit excessive inflammation and tissue damage, their suppressive activity may also hinder Th1 responses, which are crucial for bacterial control. In murine models, Mtb‐specific Tregs are among the earliest CD4 T cells to expand in the draining lymph nodes but are subsequently culled at later time points [73]. In the lung, Tregs accumulate as the infection progresses and are correlated with reduced tissue pathology [74, 75, 76, 77, 78]. Recent studies suggest that Treg‐mediated suppression in the infected lung is spatially organized, with Tregs preferentially enriched within the granuloma, alongside elevated levels of TGFβ and diminished production of IFNγ [79, 80]. Such regulatory dominance in the granuloma core may ultimately prevent bacterial clearance despite ongoing immune surveillance.

## 3 B Cells and Tuberculosis

B cells appear to play an active role in shaping the immune response to Mtb [70, 81]. In both murine and NHP models, higher numbers of pulmonary B cells are associated with reduced bacterial burden and improved disease control [82, 83]. Similarly, in humans, individuals with localized, well‐contained granulomas often display prominent B‐cell‐rich lymphoid aggregates adjacent to sites of infection [84]. These observations suggest that B cells may contribute to protective immunity either directly or by promoting the organization and function of mucosal immune responses.

### 3.1 Are B Cells Protective in TB?

During active TB, the frequency of circulating B cells decreases, while B cells aggregate within the lung parenchyma, forming organized lymphoid follicles adjacent to granulomas [85, 86]. This spatial redistribution suggests a shift from systemic circulation to more localized immune engagement, although the precise role of B cells in the lung is unclear. In humans, B‐cell depletion using rituximab has not been linked to increased TB reactivation risk [87, 88]. On the other hand, it is important to note that rituximab selectively targets CD20^+^ B cells and therefore spares plasma cells, which lack CD20 expression. It may also be less effective at depleting B cells in extravascular compartments such as the lung parenchyma [89, 90]. Nevertheless, in Mtb‐infected NHPs, B‐cell depletion has only modest effects, with a normal onset of disease despite a minor increase in bacterial burden and pathology score at the level of the granuloma [91]. Murine models have also been inconclusive, with studies of Mtb infection in B‐cell‐deficient μMT mice or after antibody‐mediated B‐cell depletion yielding a spectrum of outcomes—from modest protection to no effect or even exacerbated disease [92, 93, 94, 95, 96, 97, 98, 99]. These discrepancies underscore the complexity of B‐cell functions in TB and highlight the need for deeper characterization of their heterogeneity and antigen specificity.

A recent scRNAseq analysis of B cells isolated from human lung tissue obtained from individuals undergoing surgical resection to treat TB sequelae identified a wide variety of B‐cell subsets, including memory B cells, lung resident B cells, germinal center (GC) B cells, antibody‐secreting cells (ASC), atypical B cells (ATB), and regulatory B cells [86]. Although this study focused on total as opposed to antigen‐specific B cells, high levels of Mtb‐reactive antibodies, and in particular IgM, were detected in the infected lung. The detection of ATBs (also referred to as age‐associated B cells) suggests a possible role in TB pathogenesis. To date, ATBs have mostly been described in the context of chronic inflammatory and autoimmune diseases (e.g., malaria, sarcoidosis, lupus, multiple sclerosis) and can serve as potent antigen‐presenting cells, promote Th1 responses, support Tfh cell differentiation, or contribute to chronic inflammation during later stages of infection [100, 101, 102]. However, none of these classical B‐cell functions has been conclusively shown to be important for protection against TB. Rather, the role of B cells during Mtb infection is likely influenced by their anatomical location, the timing of their activation, and the surrounding immune environment.

### 3.2 B Cells and Antibodies

The role of antibodies in TB, particularly within the lung, has been historically underappreciated due to Mtb's intracellular lifestyle and the modest phenotype observed in B‐cell‐deficient mice [103, 104, 105]. Although μMT mice, which completely lack mature B cells, show only mild and dose‐dependent susceptibility to Mtb, mice lacking the ability to produce secreted antibodies (AID^−^/^−^μS^−^/^−^) are more susceptible to infection, indicating that under certain conditions, antibodies might contribute to protection [74]. Supporting this, both polyclonal antibodies isolated from Mtb‐exposed health care workers, but not from individuals with active TB, conferred protection in mice via immune complex formation and CD4^+^ T‐cell activation [106, 107, 108, 109, 110, 111]. Regarding antibody specificity, Mtb presents a wide range of protein and nonprotein antigens, including components of its complex mycomembrane and glycolipid capsule. The composition of these antigens can vary depending on bacterial growth and immune pressure. To date, only a limited subset of antibodies has been characterized, linking their specificity to bacterial inhibition or host protection [112, 113, 114]. Among the most widely studied Mtb surface antigens are arabinomannan (AM) and lipoarabinomannan (LAM). Polyclonal AM‐specific IgG is detectable across stages of infection [115], and BCG vaccination induces antibodies to both AM and LAM [116]. Passive transfer of monoclonal antibodies against these and other Mtb surface antigens (e.g., PstS1, LpqH) has been shown to reduce lung bacterial load in mice [117, 118]. These findings suggest that certain antibodies can contribute to protection; however, their specificity and relationship to disease state, particularly within the lung mucosa as compared to the circulation, remain incompletely defined. Moreover, antibody function is shaped not only by specificity but also by Fc‐mediated effector mechanisms. Individuals with latent TB exhibit distinct Fc glycosylation profiles compared to those with active disease, enhancing macrophage activation and inflammasome activation [119]. A recent preclinical study in mice further demonstrated that Fc‐engineered variants of an α‐glucan‐specific IgG can confer neutrophil‐dependent protection against Mtb [120]. In whole blood infection models, these protective Fc variants enhanced neutrophil activation, reduced monocyte infection, and reshaped the transcriptional profiles of innate immune cells. The emphasis of these findings is that antibody efficacy relies not only on Fab‐mediated antigen recognition but also on Fc‐effector engagement, the latter of which is influenced by the local composition of innate immune cells and their distinct Fc receptor profiles.

### 3.3 B Cells and Cytokines

B‐cell aggregation in the Mtb‐infected lung can also influence the local cytokine and chemokine landscape. In mice lacking B cells, chronic TB is associated with elevated IL‐10 and a reduction in IFNγ‐producing CD4 T cells [94, 96]. Blocking the IL‐10 receptor in these mice restores Th1 responses, suggesting that B cells promote protective Th1 effectors via their restriction of immunosuppressive IL‐10 [96]. However, the effects of selectively ablating IL‐10 in B cells have been inconsistent across studies and/or experimental endpoints: while one study reported improved host survival in mice with IL‐10‐deficient B cells, a more recent investigation found no significant impact on bacterial burden [69, 121]. In parallel, B‐cell depletion has been shown to elevate IL‐17 production and promote the accumulation of Th17 cells, leading to excessive neutrophil infiltration and heightened lung inflammation [94, 95]. Beyond IL‐10 and IL‐17, B cells also contribute to the type 1 interferon (IFN) signature observed in Mtb‐infected lungs; lung B cells from Mtb‐infected mice exhibit a type I IFN‐associated transcriptional profile, and in vitro studies confirm that B cells from both infected mice and humans secrete type I IFN. B‐cell‐derived IFN and IL‐6 can also skew macrophage polarization toward an anti‐inflammatory phenotype [122, 123, 124]. Both IL‐17 and type I IFN have been implicated in the development of TLS via their activation of lung stromal cells (discussed below), while IL‐10 may counteract this process by suppressing local inflammation and immune cell activation. Together, these findings suggest that cytokine production by B cells contributes to the formation and maintenance of lung TLS.

### 3.4 Dynamics and Location‐Specific B‐Cell Function During TB

The role of B cells in tuberculosis appears to depend on both the timing and location of their activity during infection. However, most studies investigating B‐cell function have used models of constitutive B‐cell depletion, such as μMT mice or anti‐CD20 treatment, administered prior to Mtb infection [94, 95, 99]. Moreover, as the main focus of most studies is on changes in bacterial burden, they may have overlooked the contributions of B cells to tissue pathology, a key driver of TB‐related mortality. Importantly, the timing of B‐cell depletion appears to influence the outcome of infection in an anatomic compartment‐specific manner. Early B‐cell depletion improves bacterial control in the lung‐draining lymph node, a benefit linked to reduced B‐cell expansion, preservation of stromal architecture, and enhanced CD4^+^ T cell recognition of infected myeloid cells [125]. In contrast, two separate studies using late‐stage B‐cell depletion in Mtb‐infected mice revealed distinct but complementary findings. In the first, B‐cell depletion led to increased bacterial load in the lungs, aggravated disease severity, and reduced survival [97]. The other study found that late B‐cell depletion correlated with impaired Tfh activation and mislocalization of T cells within granuloma‐associated follicles [69]. Together, these findings suggest that B cells may facilitate Mtb survival at early stages of infection but play a protective role against disease progression at later stages. Intriguingly, B cells appeared to mediate these protective effects even in the absence of canonical B‐cell functions such as antigen presentation, plasma cell differentiation, or IL‐10 production, although this interpretation is limited by the potentially incomplete efficiency of CD19‐Cre‐mediated gene deletion [69, 126, 127]. While B‐cell‐specific MHC‐II deletion did not affect bacterial burden, B cells still required cognate interactions in order to coordinate T cells positioning within granulomas, underscoring a nonclassical role for B cells in organizing cellular interactions during chronic infection.

### 3.5 Are B Cells Impaired During TB?

Although B cells are present at sites of Mtb infection, there is evidence that their function may be altered or suppressed during tuberculosis. In a 3D in vitro granuloma model generated from the PBMCs of healthy donors, selective depletion of B cells led to a significant increase in Mtb growth, supporting a potential protective role for B cells [86]. In contrast, B‐cell depletion in granuloma cultures derived from the PBMCs of individuals with active TB produced inconclusive results, suggesting that B‐cell function may already be impaired during active disease. In support of this idea, we have observed strong downregulation of both MHC‐II and PD‐L1 on lung B cells compared to their lymphoid counterparts in Mtb‐infected mice (unpublished data). This impaired phenotype persists over time and is accompanied by a striking paucity of GC B cells as well as delayed accumulation of Mtb‐specific antibody titers in the lung mucosa. Analogous disruptions have been observed during infection with *Salmonella Typhimurium* (STm), another intracellular pathogen, where B‐cell responses are predominantly extrafollicular, GC formation is absent, and antigen‐specific antibody production is limited [128]. Notably, STm infection can also extinguish pre‐existing GC responses to unrelated pathogens like influenza, highlighting the extent to which certain pathogens can override or suppress adaptive B‐cell programs [129]. Mechanistically, this has been linked to the recruitment of Sca1^+^ CX3CR1^+^ monocytes to lymphoid organs, where they induce metabolic changes in GC B cells that drive the collapse of GC reactions [129]. Given that monocytes are also actively recruited to the lungs during TB infection, it is plausible that similar monocyte‐driven mechanisms contribute to the B‐cell dysfunction observed in this setting [130]. If Mtb infection intrinsically impairs B‐cell function, then the impact of experimental interventions such as B‐cell depletion or conditional MHC‐II deletion may need to be reinterpreted, as these interventions would have little effect on an already compromised B‐cell compartment. It should be emphasized that while the delayed CD4 T cell response during TB has been recognized for decades, the idea that B‐cell function might also be delayed, suppressed, or redirected is a relatively new concept. If B‐cell responses during natural infection are indeed impaired, it raises the possibility that targeting pre‐existing B‐cell immunity—for example, through vaccination—may be a viable strategy for protection. Consistent with this idea, a recent report showed that B‐cell depletion in vaccinated NHPs abrogated protection following Mtb challenge [69].

## 4 Tertiary Lymphoid Structures

**FIGURE 1:**
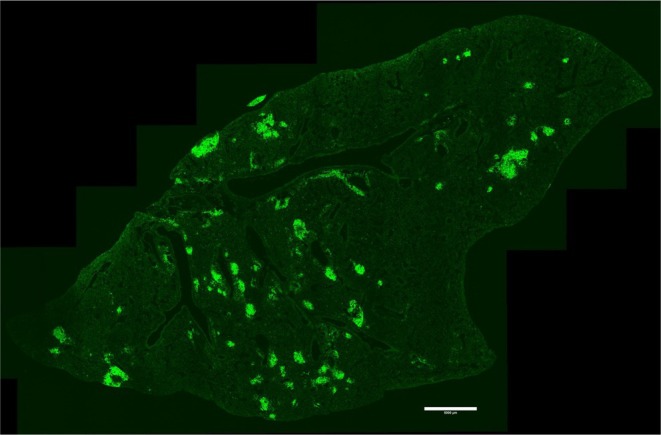
Repartition of iBALT across lung tissue. Immunofluorescence image of Mtb‐infected lung (mouse, HN878, 90 days post‐infection) stained with anti‐B220 (green). Bar, 5000 μm.

Regardless of the individual contributions made by B cells and T cells during Mtb infection, their coordinated activity within lung TLS is likely required for sustained mucosal immune responses and bacterial containment. TLS are formed in response to chronic inflammation [131, 132, 133]. They resemble secondary lymphoid organs (SLOs) in terms of cellular composition and the signaling pathways that regulate their development and function, but they are formed postnatally and typically resolve once inflammation subsides. TLS can range from loosely aggregated clusters of B and T cells with minimal organization to more mature formations containing high endothelial venules (HEVs), lymphatic vessels, dendritic cells (DCs), follicular dendritic cells (FDCs), and germinal centers (GC) [131, 134]. TLS are found in a broad range of tissues: for example, gut‐associated lymphoid tissues (GALT), nasal‐associated lymphoid tissues (NALT), and inducible bronchial‐associated lymphoid tissues (iBALT) [132]. iBALT are located within the lung parenchyma, particularly along the main bronchi, in the perivascular spaces of blood vessels, and within interstitial areas (Figure 1) [135, 136]. As the respiratory tract is continuously exposed to air, iBALT can be triggered by repeated exposure to allergens, long‐lasting particulates, and various airborne pathogens, including bacteria, viruses, and fungi [137]. iBALT development is also associated with chronic inflammatory conditions such as chronic obstructive pulmonary disease (COPD), autoimmune diseases like rheumatoid arthritis, and certain types of lung cancer [131, 138, 139, 140]. The number, size, and degree of cellular organization of iBALT vary depending on the type, duration, and magnitude of the stimulus.

### 4.1 Formation of iBALT

Unlike SLOs, iBALT forms in lung tissue that initially lacks specialized stromal cells responsible for organizing distinct T‐cell and B‐cell zones. While mature fibroblasts primarily provide anatomical support and secrete extracellular matrix, they retain a degree of plasticity and can be primed under inflammatory conditions [141]. In secondary lymph node development, lymphoid tissue‐inducer cells (LTi cells) initiate lymphoid neogenesis by promoting the differentiation of mesenchymal precursors into lymphoid‐organizing stromal cells via lymphotoxin (LT) signaling [142]. This process depends on LTβ and LTα produced by LTi cells, which signal through the LTβ‐receptor (LTβR) and TNF receptors on stromal cells. However, the formation of iBALT seems to be mechanistically distinct from lymphoid neogenesis, as mice lacking LTi cells (RORγ‐deficient and Id2‐deficient mice) still form iBALT, suggesting alternative cellular drivers such as DCs [143]. CD11c^+^ DCs produce LTβ and homeostatic chemokines such as CXCL12, CXCL13, CCL19, and CCL21. Depletion of CD11c^+^ cells leads to the disappearance or impaired formation of iBALT in models of influenza, modified vaccinia virus Ankara (MVA), and LPS exposure in neonates [143, 144, 145]. Conversely, the adoptive transfer of inflammatory bone marrow‐derived DCs (BMDCs) is sufficient to promote iBALT in naïve mice [144]. Neutrophils also contribute to iBALT induction by promoting the expression of CCL19, IL‐21, and APRIL, particularly in LPS‐exposed neonates [146]. Interestingly, a regulatory axis exists between neutrophils and Tregs: weanling mice that lack iBALT have increased numbers of Tregs and decreased numbers of neutrophils and IL‐17A‐producing cells [146]. Furthermore, Tregs also actively suppress iBALT formation at homeostasis, as CCR7‐deficient mice spontaneously develop iBALT due to defective Treg trafficking—a phenotype which is reversible by the transfer of wildtype Tregs [147]. In addition, CD206^hi^ interstitial macrophages (IMs) have recently been implicated in iBALT development. Their depletion during allergen (house dust mite) or bacterial ( *Mycoplasma pneumoniae* ) challenge markedly reduced CXCL13 expression, GC B‐cell frequency, and iBALT formation [148].

iBALT formation is additionally regulated by the production of cytokines that influence fibroblast activation and vary according to the inflammatory milieu (Table 1). During influenza infection, type I IFNs induce CXCL13 expression in a phenotypically distinct subset of pulmonary PDGFRα+ fibroblasts [149]. Similarly, repeated exposure to poly(I:C) and ovalbumin or sustained type I IFN signaling promotes the development of iBALT [150]. IL‐1 signaling is also critical, as exogenous IL‐1 administration enhances iBALT induction in influenza‐infected wild‐type mice, whereas mice lacking the IL‐1 receptor fail to develop iBALT structures [151]. IL‐17 produced by CD4^+^ T cells can also promote the expression of CXCL13 and CCL19 and is essential for iBALT initiation in LPS‐induced models [143]. While IL‐17 is required for the early initiation of iBALT in these models, it is not necessary for long‐term iBALT maintenance. Similarly, in a model using heat‐killed *P. aeruginosa* , IL‐17 deficiency leads to reduced B‐cell follicle formation, accompanied by diminished CXCL12 production by podoplanin‐expressing stromal cells, which are key organizers of the lymphoid microenvironment and essential for follicle development [153]. In *Pneumocystis* infection, IL‐13 and IL‐17 act synergistically to upregulate *Cxcl13* transcription in podoplanin^+^ stromal cells, implicating both Th2 and Th17 responses in driving iBALT development [154]. However, not all paths to iBALT‐related stromal activation require IL‐17. Intranasal administration of the MVA virus induces highly organized iBALT structures containing CXCL13‐expressing FDCs and CXCL12‐expressing follicular stromal cells through an IL‐17‐independent mechanism [153]. These findings suggest that multiple environmental cytokine signals and cellular players can converge on stromal activation to orchestrate iBALT formation.

**TABLE 1:** Inflammatory signals involved in iBALT formation and maintenance.

Soluble factors	Potential sources	Function in iBALT
Type I IFN	DCs, macrophages, stromal cells, epithelial cells, endothelial cells, B cells	Induces CXCL13 expression in fibroblasts; promote iBALT formation during influenza infection and sensitization model [149, 150]
IL‐1	DCs, macrophages, neutrophils, stromal cells, epithelial cells, endothelial cells	Enhances iBALT formation during influenza infection [151]
IL‐17	Th17 cells, γδ T cells, ILC3s	Induces CXCL13, CCL19, and CXCL12 expression in fibroblasts; essential for early iBALT formation during *Pseudomonas aeruginosa* infection, Mtb infection and LPS treatment [54, 143, 152, 153]
IL‐22	CD4 T cells, γδ T cells, ILC3s, NK cells	Promote iBALT formation during Mtb infection [152]
IL‐13	Th2 cells, ILC2s, mast cells, basophils, eosinophils	Synergizes with IL‐17 to induce CXCL13 expression during *Pneumocystis* infection [154]
CCL19/CCL21	Stromal cells, endothelial cells, DCs	Attracts T cells and DCs, necessary for T‐cell zone organization and iBALT maintenance [155, 156, 157]
CXCL13	Stromal cells, FDCs, macrophages	Attracts B cells and Tfh cells, necessary for iBALT maintenance [155, 156, 157]
CXCL12	DCs, stromal cells, endothelial cells	Attracts B cells, promote follicle formation during *Pseudomonas aeruginosa* infection [153]
LTα/LTβ	LTi cells, DCs, lymphocytes, ILC3s, NK cells	Sustains fibroblast maturation; maintains iBALT architecture [141, 144, 150]
TNFα	DCs, macrophages, stromal cells, endothelial cells, neutrophils, lymphocytes, NK cells	Sustains fibroblast maturation; maintains iBALT architecture [155]

Abbreviations: DCs, dendritic cells; IFN, interferon; ILCs, innate lymphoid cells; LT, lymphotoxin; LTi cells, lymphoid tissue inducer cells; Mtb, *Mycobacterium tuberculosis* ; NK cells, natural killer cells; Th17: Type 17 T helper cells.

During Mtb infection, the precise mechanisms underlying iBALT formation are incompletely defined. Type 3 innate lymphoid cells (ILC3s) appear to contribute by secreting IL‐17 and IL‐22 in a CXCR5‐CXCL13‐dependent manner [152]. ILC3s accumulate early in the Mtb‐infected lung, and their depletion results in a significant reduction in alveolar macrophage (AM) numbers, disruption of iBALT architecture, and impaired host resistance to infection [152, 158]. However, it remains unclear whether the effects of ILC3 depletion are direct or mediated through other cell populations. Remarkably, Mtb also modulates iBALT formation via immune evasion, with Mtb mutants lacking the membrane protein large 7 (MmpL7) or the stress response factor sigH leading to enhanced lymphoid follicle development. These findings suggest that wildtype Mtb may actively suppress iBALT formation [159, 160].

### 4.2 Maturation and Maintenance of iBALT

**FIGURE 2:**
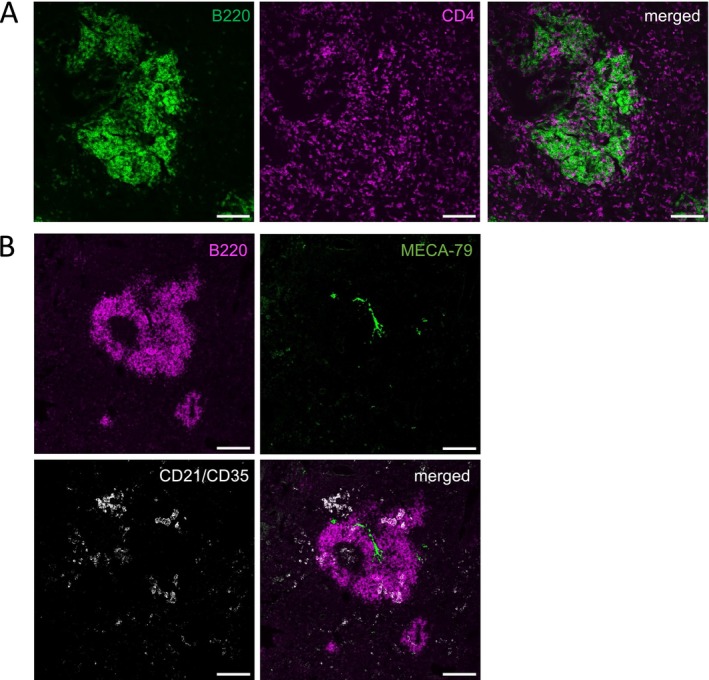
Cellular composition of iBALT in Mtb‐infected lung. (A) Immunofluorescence image of iBALT stained with anti‐B220 (green) and anti‐CD4 (magenta). Bars, 100 μm. (B) Immunofluorescence image of iBALT stained with anti‐B220 (magenta), MECA‐79 (HEVs, green), and anti‐CD21/CD35 (FDCs, white). Bars, 100 μm.

Upon activation, primed stromal cells adopt an immunofibroblast phenotype characterized by the expression of lymphoid chemokines (CCL19, CXCL13, CCL21), adhesion molecules (ICAM‐1, VCAM‐1), and survival factors (BAFF, APRIL, IL‐7) [141, 155]. The progressive recruitment of lymphocytes drives the formation of mature iBALT characterized by organized, central B‐cell follicles and peripheral T‐cell areas (Figure 2A). Although fibroblast specialization in iBALT is less well defined than in SLOs, spatial segregation of CXCL13 and CCL21 suggests the presence of B‐cell zone reticular cell (BCR)‐like and T‐cell zone reticular cell (TRC)‐like fibroblast subsets [155]. Accordingly, mice lacking CXCL13 or CCL19/CCL21 fail to establish proper B‐cell and T‐cell zones, respectively, leading to defective iBALT formation and compromised local immune responses [156, 157, 161]. Mtb and other infections, such as influenza and MVA, support mature iBALT maturation characterized by CD21/35^+^ FDCs, which support mucosal GC formation and B‐cell affinity maturation (Figure 2B) [135, 153, 156, 161]. In contrast, iBALT induced by *P. aeruginosa* lacks FDCs but contains CXCL12^+^ fibroblasts, reflecting structural and functional heterogeneity across infections and diseases [153].

Upregulation of ICAM‐1 and VCAM‐1 on fibroblasts enhances the adhesion, retention, and organization of lymphocytes within iBALT. Infiltrating lymphocytes and CD11c^+^ DCs further sustain fibroblast maturation by producing lymphotoxins (LTβ and LTα) and TNFα. Blocking LTβR signaling after iBALT is established leads to disintegration of its structure, emphasizing the essential role of fibroblasts in maintaining iBALT architecture [144, 150, 162]. Notably, iBALT can still form in the absence of LTα, suggesting partial compensation by LTβ signaling [135]. While blocking LTβR signaling is a valuable tool to study the role of iBALT in the lungs, its concomitant effect on stromal cells and endothelial cells in SLOs may limit certain interpretations [163, 164, 165]. For instance, LTβR‐Ig treatment prior to BCG infection significantly impaired granuloma formation and iNOS activity in the spleen, coinciding with increased bacterial burden and necrotic lesions. These findings indicate a critical role for LTβR signaling in macrophage activation and granuloma differentiation within lymphoid tissues; however, the observed effects may also reflect impaired early lymphocyte activation in secondary lymphoid organs due to disrupted tissue architecture and altered cytokine balance (e.g., reduced IFN‐γ and increased IL‐4) [166].

As iBALT matures, specialized vasculature, including HEVs, forms near the edges of B‐cell and T‐cell follicles (Figure 2B) [167, 168]. Analogous to SLOs, these vessels express adhesion molecules (PNAd, LFA‐1, VCAM‐1) that facilitate lymphocyte entry into iBALT [169, 170, 171]. Importantly, HEVs are not the sole entry route for blood‐derived lymphocytes into iBALT, as treatment with anti‐L‐selectin, which blocks HEV‐mediated homing, does not completely impair lymphocyte recruitment [168]. Lymphatic vessels are also present, although the organization of afferent versus efferent lymphatics remains unclear [172]. Afferent lymphatics likely transport antigens and antigen‐presenting cells into iBALT, while efferent vessels might serve as exit routes for effector lymphocytes to access draining lymph nodes and the circulation. Impaired drainage from pulmonary lymphatics can be an additional trigger for iBALT formation, and in naïve mice, lymphatic obstruction is sufficient to induce iBALT structures in the absence of exogenous antigens [173]. Similarly, disruption of CCR7‐dependent DC migration from the lung to the draining lymphatics leads to spontaneous iBALT formation [168]. These findings suggest that the local accumulation of immune cells within the lung parenchyma, whether due to persistent chemotactic signaling or a lack of egress options, can contribute to the initiation and organization of iBALT structures.

### 4.3 iBALT Function

The impact of iBALT on lung immunity is highly context‐dependent; it can have either protective or pathogenic effects depending on the underlying inflammatory conditions [174]. In infectious settings, iBALT supports robust local adaptive immune responses and can support de novo lymphocyte priming, mucosal antibody production, and memory formation [131]. Remarkably, mice lacking SLOs but possessing iBALT are capable of clearing the influenza virus and mounting effective recall responses [135]. These structures support the long‐term maintenance of memory CD8^+^ T cells and antigen‐specific antibodies [167]. Influenza‐induced iBALT contains GC B cells expressing canonical markers (e.g., *Bcl6*, *Fas*, *Aidca*, *S1pr2*), as well as Tfh cells and FDCs, facilitating local affinity maturation and class‐switch recombination [175].

**FIGURE 3:**
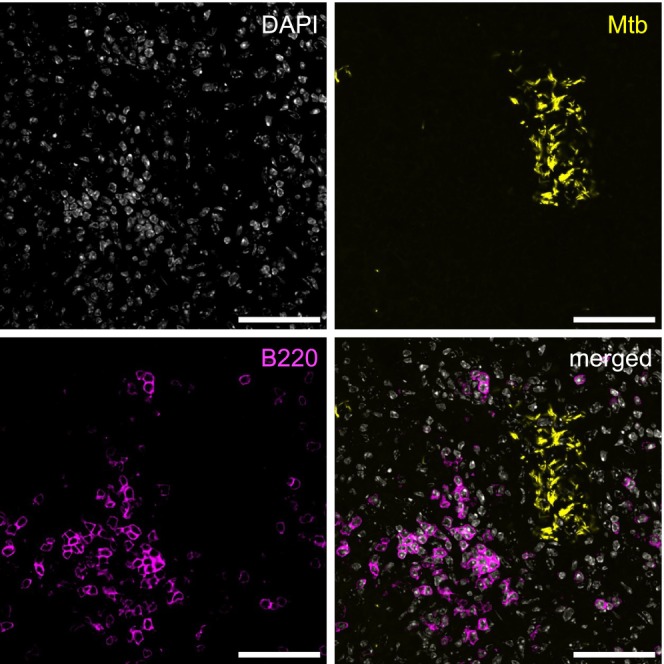
iBALT forms near sites of Mtb bacilli and infected cells in mice. Immunofluorescence image of Mtb‐infected lung with a reporter strain (Turbo635‐HN878) stained with anti‐B220 (magenta) and co‐labeled with DAPI (white). Bars, 50 μm.

iBALT has also been observed in response to bacterial and fungal pathogens, with subtle differences in signaling pathways and cellular composition [153, 154, 176, 177]. However, the precise contribution of iBALT in these settings remains incompletely understood. In chronic infections with *Pseudomonas aeruginosa* and *Staphylococcus aureus* , both commonly found in patients with cystic fibrosis, iBALT formation is induced in murine models and is associated with peribronchial lymphoid neogenesis and progressive lung damage in humans [178]. In TB, iBALT localizes at the peripheral rim of granulomas in both humans and NHPs, and forms near sites of Mtb bacilli and infected cells in mice (Figure 3) [83, 84, 159]. Their increased size and organization during latent infection, compared to active disease, suggest a potential role as morphological correlates of protective immunity [70, 81]. However, it remains unclear whether iBALT forms as a result of immune control or actively contributes to protection.

Lung TLS have also been observed in solid tumors and are generally associated with improved prognosis, including in non‐small‐cell lung cancer [131, 138, 179]. These structures support antitumor immunity by facilitating DC‐mediated antigen presentation, activation of effector T cells, and local antibody production [180, 181]. TLS density and cellular composition—typically including CD4^+^ and CD8^+^ T cells, follicular B cells, mature DCs, and HEVs—serve as predictive biomarkers for response to immunotherapies. Notably, their presence in the lung has been associated with enhanced efficacy of immune checkpoint blockade [182, 183]. However, in certain cases, TLS may promote tumor progression by fostering immunosuppressive microenvironments [138]. For instance, in lung adenocarcinoma, TLS enriched with Tregs can suppress CD8^+^ T cell activity, and Treg depletion has been shown to facilitate tumor regression [184].

iBALT has been implicated in the pathogenesis of chronic lung diseases such as COPD, rheumatoid lung disease, hypersensitivity pneumonitis, and asthma [139]. Its presence is often correlated with more severe disease phenotypes, indicating a potential role in exacerbating these conditions. In murine models of cigarette smoke exposure, disruption of iBALT has been shown to promote lung tissue regeneration and reverse airway fibrosis [162]. In allergic airway disease, repetitive antigen exposure drives the formation of ectopic GC reactions within the lung, promoting somatic hypermutation, affinity maturation, and the local generation of tissue‐resident memory B cells [185]. Yet, the role of iBALT in allergic sensitization remains ambiguous. While iBALT supports the maintenance of pathogenic memory Th2 cells, pre‐existing iBALT in sensitized mice has been shown to delay Th2 cell accumulation, reduce eosinophilic infiltration, and attenuate lung inflammation [186, 187]. In autoimmune lung diseases, iBALT contributes to the local production of autoreactive B cells and autoantibodies, correlating with disease severity [140, 188]. Similarly, in lung transplantation models, the *de novo* formation of lymphoid structures around intrapulmonary tracheal allografts is associated with local alloimmune activation and an increased risk of graft rejection [189, 190].

### 4.4 T‐B Cell Interactions

Spatial co‐localization of T cells and B cells within iBALT supports the formation of ectopic GC reactions, promoting local antibody production and the generation of tissue‐resident memory B cells [191, 192, 193, 194]. Tissue‐resident T helper (TRH) cells engage naïve B cells expressing CD40, PD‐L1/PD‐L2, ICOSL, and IL‐21R, promoting B‐cell activation and differentiation. Following viral infection, TRH cells arise independently of lymph node‐derived Tfh cells and require both B cells and intrinsic Bcl6 expression for their maintenance [192]. While early, transient Bcl6 expression supports CD4 T cell expansion, sustained expression is required for their retention within iBALT and for effective mucosal immunity. Late deletion of *Bcl6* in CD4 T cells impairs their retention in iBALT, leading to diminished mucosal antibody production and increased viral load.

During Mtb infection, CXCR5^+^ CD4 T cells localize within lymphoid follicles in the lungs of humans, NHPs, and mice [70]. These cells co‐express ICOS and PD‐1 and produce proinflammatory cytokines such as IFNγ, TNFα, IL‐2, IL‐17, and IL‐21. Complementary findings from human TB lungs show subsets of B cells expressing CCR7 and CD40, suggesting their capacity to migrate toward T‐cell zones and participate in T‐B cell interactions [86]. In mice, Mtb‐specific PD‐1^+^ KLRG1^−^ CD4 T cells have been identified in the lung parenchyma and exhibit Tfh‐like features, including expression of CXCR5, ICOS, and Bcl6 [71]. More recent scRNAseq analysis further revealed the persistence of a Tfh‐like population in the lung at both 6 and 41 weeks postinfection [46]. While initially polyfunctional, this subset exhibits a progressive loss of *Tnf* and *Il21* expression over time, suggesting exhaustion or a shift in effector capacity. Importantly, in influenza‐infected lungs, TRH cells retain the ability to differentiate into Th1 effectors and express high levels of Tcf1, a transcription factor associated with self‐renewal and stem‐like properties [192]. In the case of TB, these findings raise the possibility that iBALT‐associated T‐B cell interactions may support a replenishment of Th1 cells within the lung microenvironment.

Further emphasizing the role of Tfh cells in TB disease, mice lacking *Bcl6* expression in CD4 T cells exhibit increased Mtb burden and reduced survival [69]. However, *Bcl6* is also transiently expressed during early CD4 T cell priming and contributes to T‐cell expansion, including tissue resident Th1 cells [192, 195, 196]. In an influenza model of infection, inducible deletion of *Bcl6* in CD4 T cells, after iBALT formation, led to an emigration of CD4 T cells away from B‐cell clusters. This reduced colocalization of CD4 T cells and B cells was functionally relevant as mucosal antibodies were decreased following heterologous challenge. Colocalization of CD4 T cells and B cells is also relevant during Mtb infection, where PD‐L1/PD1 interactions regulate Tfh positioning and activation [69]. In Rag1‐KO mice, which lack mature B and T cells, co‐transfer of wild‐type B cells with *Pdcd1*‐KO T cells led to a marked reduction of IFNγ^+^ Tfh‐like cells and disrupted lymphoid follicle architecture. These findings indicate a potential role for B‐cell intrinsic PD‐L1 expression in T‐cell positioning within iBALT. However, it is worth noting that PD‐L1 is also upregulated on myeloid and stromal cells during Mtb infection, suggesting that multiple cell types may contribute to the regulation and spatial positioning of Tfh cells within iBALT.

## 5 TLS: Inside out Signaling

Although iBALT has traditionally been associated with local antibody production, it may also function as a site for T‐cell priming, providing an alternative to classical activation in SLO, which can undergo structural disruption during Mtb infection. T‐cell responses are typically initiated in draining lymphoid organs, where antigen recognition and costimulation trigger activation and expansion, followed by migration to peripheral tissues and effector differentiation. This sequence of activities is referred to as an “inside‐out” immune response, in which adaptive immunity originates in the SLO and is executed in the tissue [197]. iBALT has been shown to function as a site for de novo T‐cell recruitment and priming, effectively recapitulating the “inside‐out” model within a single organ. An example is seen during influenza infection, where heterogeneous lung‐resident CD4 T cell subsets, including TRH and Th1 cells, are found in and around iBALT [192, 198]. Notably, TRH cells, which depend on the presence of B cells, comprise two subsets regulated by the transcription factors Bcl6 and HIF‐1α, respectively, pointing to B‐cell‐dominated iBALT as an important site for local T‐cell diversification.

Our recent work reveals compartmentalization within iBALT, with gene signatures associated with GC B cells and Tfh cells centralizing in the iBALT core, and signatures related to T‐cell receptor signaling, hypoxia, and TGFβ predominating in the iBALT periphery [199]. Both hypoxia and TGFβ pathways are known to support HIF‐1α activity in T cells [200, 201, 202], and HIF‐1α‐active CD4 T cells are accordingly detected by immunofluorescence at the iBALT periphery. This spatial organization suggests a functional division between Tfh‐like TRH cells in the core and HIF‐1α + TRH cells at the iBALT borders. Inducible deletion of *Hif1a* in CD4 T cells, at the onset of its activity in the lung but after initial T‐cell priming in SLO, led to reduced numbers of Th1 cells, alveolar macrophages, and lung‐resident NK cells, as well as diminished influenza‐specific IgA titers. Importantly, these effects, extending out into the lung from the edge of iBALT structures, occurred in the absence of apparent alterations to peripheral T‐cell responses. These findings identify a distinct HIF‐1α + CD4 T cell population associated with iBALT that orchestrates a coordinated network of local immune responses engaged during viral infection.

In light of these findings, a similar HIF‐1α‐dependent mechanism may operate during TB, not only shaping local CD4 T cell responses but also orchestrating a broader immune cell network involving NK cells, alveolar macrophages, and antibodies. In TB, granulomas develop hypoxic cores as they progress toward necrosis, with oxygen tension decreasing toward the center [203]. This hypoxic environment leads to stabilization of HIF‐1α in multiple cell types, including macrophages, neutrophils, and T cells. HIF‐1α plays a critical role in regulating immune responses under low oxygen by promoting glycolytic metabolism and inflammatory responses [204]. However, its role in TB is controversial. While early HIF‐1α activity supports host defense, elevated HIF‐1α expression has also been associated with enhanced Mtb survival during chronic infection [205]. Additionally, the specific function of HIF‐1α in T cells is not fully understood in the setting of TB. Constitutive HIF‐1α activity in T cells, achieved through *Vhl* deletion, led to increased susceptibility to Mtb infection [206]. These HIF‐1α‐stabilized T cells exhibited impaired activation, characterized by reduced expression of activation markers and diminished IFN‐γ production—defects that were reversed upon *Hif1a* deletion. However, modulation of HIF‐1α activity at the onset of infection may impair early T‐cell differentiation in the lymph nodes. Therefore, temporally controlled modulation of HIF‐1α, particularly at later stages of infection, may be a more appropriate strategy for evaluating tissue‐resident T‐cell responses.

While evidence from Mtb infection models is currently lacking, tissue resident memory T cells (TRM) additionally show the ability, when reactivated, to leave their home tissue and re‐enter the circulation and SLO [207]. This “outside in” potential of TRM deserves further scrutiny in TB animal models because of the multiorgan nature of the infection and the established risk of progression from contained, latent infection to active disease.

T‐cell trafficking of this kind exemplifies another major function of TLS: as a node in the lymphatic vasculature, which along with the circulatory system, provides the infrastructure for immune cell surveillance and migration throughout the body. iBALT typically forms along blood vessels and lymphatic tracts, establishing a strategic anatomical niche for immune cell trafficking in and out of the tissue. In Mtb‐infected lungs, inflammatory foci are frequently located in proximity to LYVE‐1+ lymphatic endothelium [208]. Infection promotes lymphangiogenesis, characterized by lymphatic vessel sprouting and an overall expansion of the lymphatic network in the lung. This process is regulated by the expression of vascular endothelial growth factor receptor 3 (VEGFR3) on LECs and persists even after the resolution of acute infection [208]. Furthermore, mature iBALT structures are often characterized by de novo formation of HEVs. Despite the anatomic proximity between iBALT and the vascular system, the potential contribution of blood and lymphatic endothelial cells remains underexplored in the study of TB.

### 5.1 Dynamic Lymphatics

The lymphatic vasculature plays a central role in immunity by mediating the bidirectional transport of antigens, immune cells, and inflammatory signals between peripheral tissues and draining lymph nodes [209]. During inflammation, vascular permeability is modulated by proinflammatory mediators, enhancing the infiltration of immune cells into the interstitium. LECs dynamically interact with the immune response in several ways [210]. They produce a range of chemoattractants—including CXCL12, CX3CL1, CCL2, CXCL8, CCL21, and CCL19—that guide the migration of DCs, macrophages, neutrophils, and lymphocytes expressing the corresponding receptors (CXCR4, CX3CR1, CCR2, CXCR1/CXCR2, CCR7). LECs also express key adhesion molecules, including PECAM‐1, ICAM‐1, VCAM‐1, CD99, and selectins, which facilitate leukocyte rolling, adhesion, and transmigration. LECs upregulate MHC‐II in response to inflammation, suggesting a potential role in antigen presentation [211]. While direct presentation of antigen by LECs to CD4 T cells remains unclear, current evidence indicates that they may contribute to this process at least indirectly, through cooperation with DCs [212, 213]. The presence of lymphatic vessels in close proximity to granuloma‐associated iBALT may provide a direct entry route for immune cells to access sites of T‐B cell interaction and coordination.

Naive B and T cells circulating in the blood enter the lymph nodes primarily through HEVs, a specialized subset of postcapillary venules adapted for large‐scale leukocyte trafficking [170]. Upon entry into the lymph node, T cells migrate to the paracortical T‐cell zones, while B cells localize to the follicles within the cortical region. This compartmentalized migration is directed by a finely tuned chemokine network: fibroblastic reticular cells (FRCs) in the T‐cell areas produce CCL19 and CCL21, which attract CCR7^+^ T cells, whereas FDCs in the B‐cell follicles secrete CXCL13 to recruit CXCR5^+^ B cells and Tfh cells. In addition to lymphocytes, HEVs facilitate the entry of plasmacytoid dendritic cells (pDCs) and precursors of conventional dendritic cells (pre‐cDCs) [214, 215]. Notably, DCs are often positioned adjacent to HEVs, where they influence endothelial cell function by producing lymphotoxins (LTα, LTβ, LIGHT) [216]. These ligands engage LTβR on endothelial cells, contributing to HEV maturation and maintenance. Given the structural and functional similarities between iBALT and lymph nodes, the de novo formation of blood vessels at the periphery of iBALT may provide an efficient entry point for circulating naïve lymphocytes, supporting mucosal T‐ and B‐cell priming and thereby reinforcing the role of iBALT as a functional site of adaptive immune activation.

Lymphatic vessel expansion plays a crucial role in enhancing immune cell trafficking and tissue repair, so it is important to elucidate the underlying mechanisms of lymphangiogenesis in TB. This process involves the emergence of specialized “tip cells” that guide new vessel growth, under the control of VEGFR3 signaling [217]. While replication of resident LECs is a primary mechanism of postembryonic lymphangiogenesis, circulating progenitor cells, and in particular, myeloid‐derived and stromal cells, can also contribute [17, 218, 219]. Both cell types support lymphatic growth either by secreting pro‐lymphangiogenic factors or by transdifferentiating into LECs and merging directly into growing vessels. In models of wound inflammation, CD11b^+^ cells co‐expressing LEC markers (podoplanin, LYVE‐1) have been observed within and around lymphatic vessels [220]. Similarly, CD11b^+^ alveolar macrophages isolated from patients with idiopathic pulmonary fibrosis demonstrated the capacity to form lymphatic‐like structures ex vivo [221]. In the tumor microenvironment, tumor‐associated macrophages have been shown to acquire a lymphangiogenic phenotype in response to TNFα signaling [222]. As noted by Gutierrez and colleagues, and considering the role of TNFα in granuloma formation [223], it will be important to investigate whether macrophages within granulomas contribute to lymphangiogenesis during Mtb infection [17].

### 5.2 Lymphatics: Pathway to Mtb Persistence?

The expansion of blood and lymphatic vessels at sites of Mtb infection facilitates immune cell trafficking to and from the lung. However, this vascular remodeling may also contribute to bacterial persistence. Mtb has been shown to replicate within LECs, indicating that lymphatic vessels can serve as a permissive niche for the pathogen [224]. Supporting this, aerosol infection in guinea pigs revealed pulmonary lymphatics as primary sites of infection [225]. These findings suggest that lymphatic vessels may provide a direct route for Mtb from the lung parenchyma to draining lymph nodes. Interestingly, similar mechanisms are observed in cancer, where increased lymphatic vessel invasion is associated with a higher risk of lymph node metastasis and tumor spread [226, 227, 228].

In the opposite direction, the lymphatic system may enable the re‐entry of Mtb from infected lymph nodes or other sites into the lung, leading to the development of secondary lesions and possible reactivation of disease. Indeed, Mtb infection is increasingly recognized as a lymphatic disease [16, 17]. Behr and colleagues proposed a model in which Mtb initially transits asymptomatically through the lung and establishes a chronic infection within the lymph nodes [16]. The anatomical separation between the primary lesion and sites of reactivation, typically in the lung apex, suggests that disease reactivation may involve lymphatic‐mediated reseeding of the pulmonary parenchyma. Strikingly, autopsy studies have identified tuberculous lymphadenopathy in individuals who died from unrelated causes and showed no evidence of pulmonary tuberculosis, indicating that latent infection can persist in lymph nodes independently of active lung disease [229].

### 5.3 Lymphatic Perturbation as Therapy

Lymphangiogenesis has also been observed during influenza infection, characterized by a transient yet substantial increase in LEC numbers and vessel extension, peaking at 14 days postinfection [230]. In this model, specific deletion of *Pd‐l1* in LECs (*Pd‐l1* ^loxp/loxp^ x *Prox1*‐CreERT2) resulted in enhanced lymphangiogenesis, implicating PD‐L1 as a negative regulator of lymphatic vessel expansion. Interestingly, similar observations were made in the lymph nodes, where type I IFN signaling promotes PD‐L1 expression in LECs and inhibits their division [231]. While the increase in lymphatic density did not impact lung pathology during influenza infection, it raises important questions about the potential consequences of using a mouse model of specific *Pd‐l1* deletion in LECs as a modulator of lymphangiogenesis in Mtb infection.

Enhancing lymphatic vessel density in Mtb‐infected lungs may have both beneficial and detrimental consequences. It may support protective immunity by facilitating the trafficking of antigen‐presenting cells from the lung to draining lymph nodes, along with the recruitment and local priming of naive T cells in the lung. However, it may also expand a permissive niche for Mtb and facilitate its spread throughout the lymphatic network. This tension underscores a central challenge in TB: balancing immune responses that cause inflammation and tissue damage with responses that support bacterial clearance.

Evidence from other disease models supports the potential of modulating local immune structures to improve host outcome. For example, in a murine tumor model, treatment with anti‐PD‐L1 antibody increased the number, size, and organization of iBALT, correlating with improved tumor control [232]. However, therapeutic strategies targeting the PD‐1/PD‐L1 axis may have unintended consequences when applied to infectious diseases. In Mtb infection, PD‐1 knockout mice have worse disease outcomes, and immune checkpoint blockade in Mtb‐infected humans and nonhuman primates has been linked to TB reactivation or exacerbation [233, 234, 235]. These divergent effects likely reflect the complex and context‐specific roles of PD‐1/PD‐L1 signaling across different cell types and disease states. PD‐1 can be expressed not only by T cells but also by B cells, dendritic cells, ILCs, and NK cells. Similarly, PD‐L1 is expressed on a wide range of immune cells, including T cells, B cells, dendritic cells, macrophages, neutrophils, as well as nonimmune structural cells, including fibroblasts, epithelial cells, and endothelial cells. Accordingly, more refined approaches such as temporally regulated and cell type‐specific genetic models are necessary to disentangle the potential benefits of immune modulation while minimizing potential harm.

PD‐L1 expression on LEC may also act as an immunoregulatory mechanism by modulating T‐cell activation within the lung. Under steady state conditions, lymph node‐resident LECs contribute to peripheral tolerance by presenting self‐antigen via MHC‐I and expressing PD‐L1, thereby inhibiting autoreactive T‐cell responses [209]. During inflammation, PD‐L1 expression on LECs is upregulated in various settings, including tumor models and contact hypersensitivity [236, 237]. Our preliminary data further indicate that PD‐L1 is upregulated on lung‐resident LECs following Mtb infection (unpublished data). In line with this, lymphatic‐specific deletion of PD‐L1 in a tumor model led to enhanced CD8 T cell responses, highlighting the potential of LECs to modulate local T‐cell immunity [238]. Interestingly, PD‐1/PD‐L1 interactions between LECs and T cells have also been implicated in facilitating the transmigration of Tregs and CD4 effector T cells across the lymphatic endothelium [239]. These findings suggest that PD‐L1 expression by LECs may contribute to immune regulation in Mtb‐infected lungs.

## 6 TLS: Are They Always Good?

While iBALT formation is often associated with host protective immune responses in infection and tumor models, it can also contribute to disease pathology in chronic inflammatory settings. For instance, in a mouse model of COPD, elevated expression of LTβR ligands promotes epithelial cell apoptosis via activation of the noncanonical NFκB pathway, leading to emphysema, collagen deposition, and muscle wasting, which could be reverted by blocking LTβR signaling [162]. In allergy, iBALT structures promote persistent lung inflammation by preserving pathogenic memory Th2 cells through a mechanism involving IL‐7‐producing LECs [186]. These examples underscore that iBALT in TB might be viewed not solely as a marker of protective immunity, but rather as an indicator of ongoing immune responses, the consequences of which depend on the nature and regulation of the surrounding immune environment.

Most studies investigating iBALT define B‐cell affinity maturation and mucosal antibody production as key features of protective immune responses. Our own work additionally highlights the importance of iBALT in orchestrating tissue‐localized inside‐out signals, which contribute to a broader network of mucosal immune activity, including alveolar macrophages and NK cells [199]. However, the full spectrum of iBALT functions in TB remains unclear, and it is possible that they play additional, yet undiscovered, roles in shaping local immune and tissue responses. For example, findings from a mouse model of colitis showed that type I interferon signaling drives the expansion of a population of B cells expressing Sca‐1 and PD‐L1 during the recovery phase [240]. Increased frequencies and numbers of these B cells were associated with greater body weight loss and more severe disease. Notably, B cells were observed forming TLS in close proximity to the intestinal epithelium, thereby increasing the distance between fibroblasts and epithelial cells and disrupting their interaction. In this model, scRNAseq revealed that B‐cell depletion enhanced mucosal healing and altered gene expression related to tissue remodeling in both stromal and epithelial cells. These findings suggest a potential role for B cells in modulating stromal cell responses that contribute to tissue pathology during chronic inflammation. B cells have also been shown to influence stromal cell remodeling within lymph nodes, with divergent outcomes depending on the infectious context. During helminth infection, B‐cell‐derived lymphotoxin signaling promotes the expansion of FRC numbers and reorganization of the paracortex, leading to the formation of new B follicles [241]. These structural changes support enhanced antibody production and reduced worm burden. In contrast, B‐cell expansion within *Mtb*‐infected lymph nodes drives the translocation of follicles into the paracortical region, disrupting the organization of the CCL21^+^ FRC network [125]. This remodeling impairs T cell access to Mtb‐infected CD11b^+^ cells, thereby compromising efficient T‐cell priming and resulting in increased lymph node bacterial burden. Together, these findings highlight a role for B cells in shaping stromal cell phenotype within the intestinal and lymph node microenvironments and raise important questions about how B‐cell–stromal cell interactions might influence lung pathology during TB. Given the central role of stromal cells in tissue repair and remodeling, it is possible that similar immune‐stromal dynamics in the TB‐infected lung contribute to pathologic outcomes such as fibrosis.

Pulmonary fibrosis is a well‐recognized hallmark of TB pathology, reflecting a maladaptive wound‐healing response characterized by excessive extracellular matrix deposition. This process leads to stiffening of the lung parenchyma, architectural distortion, and the formation of fibrotic scars that impair respiratory function [242]. Granulomas can evolve into fibrocaseous or fibrocalcific lesions, encased by collagen‐rich rims or containing extensive intralesional fibrosis, resulting in permanent damage that persists even after successful antibiotic treatment [243, 244]. Many individuals recovering from TB experience persistent pulmonary dysfunction, underscoring the lasting impact of fibrotic scarring and dysregulated tissue repair in post‐TB lung disease [245, 246, 247, 248].

TLS have been implicated in the progression of fibrosis and tissue damage in several age‐related and autoimmune diseases [131]. Acting as persistent inflammatory hubs, TLS can sustain chronic pro‐inflammatory cytokines, creating a microenvironment that negatively impacts surrounding parenchymal cells. In lupus nephritis, TLS formation in the kidney is associated with poor prognosis and contributes to disease pathogenesis [249]. In aged‐mouse kidneys after ischemia–reperfusion injury, profibrotic VCAM‐1+ proximal tubular cells preferentially localize near TLS and promote fibroblast activation via TGFβ signaling [250]. Additionally, Tfh cells within TLS contribute to renal fibrosis by secreting IL‐21 [251]. In systemic sclerosis, hyperactive and dysregulated B cells accumulate within pulmonary TLS and are linked to ongoing fibrotic remodeling, in part by supporting the differentiation of profibrotic macrophages [252, 253]. In IgG_4_‐related disease, a fibroinflammatory condition, B cells isolated from patients produce profibrotic factors and stimulate collagen production by fibroblasts [254]. Collectively, these examples illustrate that in certain inflammatory environments, TLS and B cells can actively contribute to tissue fibrosis and organ dysfunction.

In light of these findings, it is critical to investigate both iBALT and B cells in modulating stromal cell responses during Mtb infection in the lung. Our preliminary data in Mtb‐infected mice show that while both late‐stage B‐cell depletion and TLS disruption lead to a more disseminated distribution of bacteria in the lung, they have distinct effects on stromal cell activation and immune cell composition (unpublished data). Notably, B‐cell depletion resulted in increased lung fibrosis consistent with findings from another study [97]. However, we did not observe fibrosis following TLS disruption. These results indicate that B cells may restrain fibrotic remodeling through mechanisms that are independent of their role in TLS.

A deeper understanding of how stromal cells modulate immune and tissue responses in the Mtb‐infected lung is required. Fibroblasts, which are present in both iBALT and granulomas, play a key role in organizing immune cell compartmentalization and forming physical barriers that isolate infected tissue from healthy tissue, contributing to bacterial containment. However, fibroblast functions can have detrimental consequences. In granulomas, fibroblast‐derived collagen rims may restrict effector T cell access to Mtb‐infected cells and limit antibiotic penetration, potentially hindering bacterial clearance. Moreover, maladaptive fibroblast activation can lead to excessive extracellular matrix deposition and fibrotic scarring, resulting in long‐term structural damage and impaired lung function.

## 7 Conclusions

iBALT has drawn increasing attention in pulmonary disease research for its role in coordinating localized immune responses within affected tissues. In TB, histological analyses of infected lungs reveal prominent B‐cell‐rich lymphoid aggregates near sites of infection, particularly during latent stages, where their presence is associated with better disease control. However, the specific mechanisms by which iBALT may contribute to protective immunity in TB remain incompletely understood. Drawing parallels from influenza models, iBALT may facilitate mucosal antibody production and support T‐cell survival and diversification, potentially enhancing immune access to granulomatous lesions. However, the contribution of antibodies to TB immunity is still unclear, and the role of iBALT in shaping T‐cell function requires further investigation. In addition to its potential protective roles, TLS and B‐cell responses can also trigger fibrosis and dysregulated healing. Post‐TB tissue remodeling thus remains an important and underexplored area. Understanding how iBALT arises, persists, and functions during Mtb infection could provide valuable insights to advance therapeutic and prophylactic strategies against pulmonary TB.

## Conflicts of Interest

All the authors declare no conflicts of interest related to this review.

## Data Availability

The authors have nothing to report.
